# Identification of QTLs Associated with Stem Breaking Strength and Development of InDel Markers in Soybean Using BSA-Seq

**DOI:** 10.3390/plants15111610

**Published:** 2026-05-24

**Authors:** Piao Leng, Kelin Deng, Jiangang An, Wenying Yang, Jianqiu Liang, Jun Feng, Haiying Wu, Longxi Zhang, Li Liu, Haifeng Chen, Xiaobo Yu, Zhaoqiong Zeng

**Affiliations:** 1 Nanchong Academy of Agricultural Sciences, Nanchong 637000, China; lengpiao1997@163.com (P.L.); ajgang@126.com (J.A.); yangwenying1315@163.com (W.Y.); liangjianqiu142@163.com (J.L.); 13990750512@163.com (J.F.); ncnkswhy@sina.com (H.W.); bo0524@163.com (X.Y.); 2 Oil Crops Research Institute, Chinese Academy of Agricultural Sciences, Wuhan 430062, China; 18369696664@163.com (K.D.); chenhaifeng@caas.cn (H.C.); 3 Nanchong Economic Crop Management Station, Nanchong 637000, China; z18802466438@163.com; 4 Nanchong Seed Administrative Station, Nanchong 637000, China; 18781738635@163.com

**Keywords:** *Glycine max*, stem breaking strength, lodging resistance, BSA-Seq, QTL-seq, InDel marker, candidate gene

## Abstract

Stem lodging significantly reduces soybean yield stability, particularly under dense planting and intercropping systems. Stem breaking strength is a key component of lodging resistance, but its genetic basis remains incompletely understood. In this study, an F_2_ population consisting of 167 individuals derived from a cross between nanxiadou25 (NXD25, high stem breaking strength) and Shiyuehuang (SYH, low stem breaking strength) was analyzed using bulked segregant analysis with whole-genome resequencing (BSA-Seq) to identify loci associated with stem breaking strength. The trait showed broad quantitative variation in the F_2_ population, ranging from 20.1 to 673.7 N. Two extreme bulks were constructed using 30 plants with the highest values and 30 plants with the lowest values. QTL-seq detected 21 candidate intervals at the 95% confidence level, among which, three major loci on Chr07, Chr13, and Chr16 exceeded the 99% threshold and were designated qBR7.2, qBR13.1, and qBR16.1. By integrating large-effect SNP/InDel variation, marker development, RNA-seq profiling, and qRT-PCR validation, nine candidate genes were retained for further study, and three marker-linked genes were highlighted as high-priority candidates. RNA-seq identified 9617 differentially expressed genes between the two parents. In addition, three co-dominant InDel markers, Chr07_01, Chr13_17, and Chr16_83, showed phenotype-consistent polymorphism in extreme F_2_ individuals. These findings provide valuable loci, candidate genes, and molecular markers for soybean lodging-resistance breeding.

## 1. Introduction

Soybean (*Glycine max*) is one of the world’s most important oil and protein crops and is central to global food and feed supply [1]. Lodging remains a major constraint on soybean yield stability, particularly under dense planting conditions, where it can reduce harvestable yield by 10–30% and increase mechanical-harvesting losses [2]. Stem breaking strength is a key component of lodging resistance and is therefore an important target for genetic improvement [3].

Although Green Revolution strategies improved lodging resistance in rice and wheat by reducing plant height [4], this approach is less straightforward in soybean because excessive height reduction may decrease pod number and final yield [5,6]. Therefore, improving stem mechanical strength while maintaining productivity is a practical breeding objective [7,8]. At the anatomical level, lodging resistance is associated with sclerenchyma and collenchyma development, vascular bundle organization, cortical compactness, and cell wall composition [9,10]. Lignin and cellulose, in particular, contribute to stem rigidity and resistance to breakage [11].

Bulked segregant analysis combined with whole-genome sequencing (BSA-Seq) provides a rapid and cost-effective approach for mapping quantitative trait loci (QTLs) associated with complex agronomic traits [12,13]. In QTL-seq analysis, individuals with extreme phenotypes are pooled, resequenced, and compared based on allele-frequency differences [14]. This strategy has been widely used in crops and is especially useful for preliminary mapping of major-effect loci in segregating populations [15,16].

NXD25 is an elite lodging-resistant line with high stem breaking strength, high yield, and wide adaptability. SYH is a local landrace with low stem breaking strength and weak lodging resistance. In this study, an F_2_ population derived from the cross NXD25 × SYH was used to dissect the genetic basis of stem breaking strength in soybean [17,18,19]. The objectives were to identify major loci, develop InDel markers, and prioritize candidate genes for marker-assisted breeding [20,21].

## 2. Results

### 2.1. Stem Histological Differences Between the Two Parents

**Figure 1:**
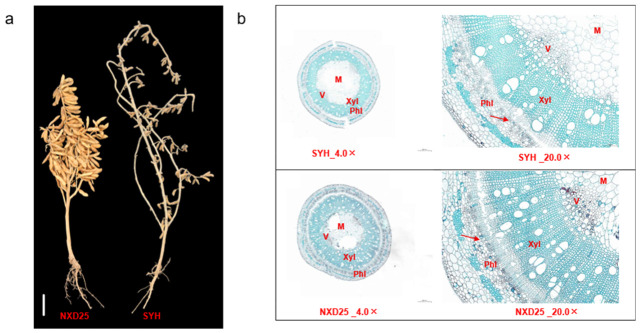
Phenotypic and histological comparison of NXD25, SYH, and representative F_2_ individuals. (**a**) Whole-plant morphology of NXD25 and SYH and representative stem cross sections at 4.0× and 20.0× magnification. (**b**) Enlarged histological comparison of stem cross sections between NXD25 and SYH. Xyl, xylem; Phl, phloem; V, vascular bundle region; M, pith.

Paraffin cross-section analysis showed clear histological differences between the two parents. NXD25 displayed thicker peripheral mechanical tissues, more developed vascular bundles, and a more compact cortical structure, whereas SYH showed thinner supportive tissues, less-developed vascular tissues, and a looser basic tissue organization (Figure 1). These stem-section differences are consistent with the observed variation in bending resistance and overall stem mechanical performance, suggesting that stronger stem support in NXD25 is closely related to enhanced development of mechanical and vascular tissues.

### 2.2. Phenotypic Variation in Stem Breaking Strength

**Figure 2:**
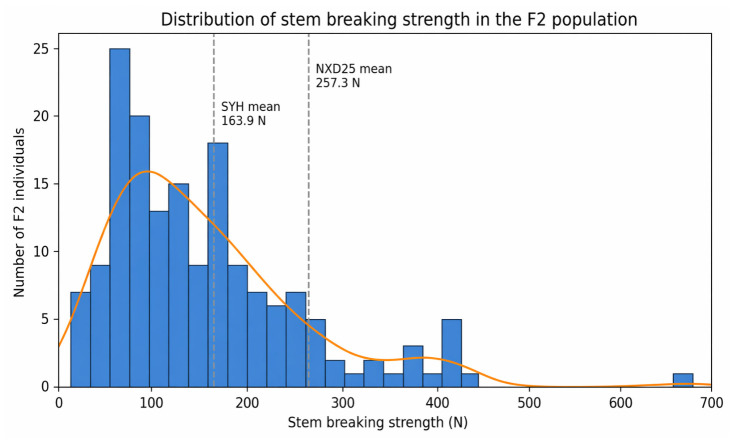
Frequency distribution of stem breaking strength in the F_2_ population. The histogram shows the continuous distribution of the trait in 167 F_2_ individuals, and dashed vertical lines indicate the parental mean values of NXD25 and SYH.

Using the recovered phenotyping dataset, mean stem breaking strength was 257.3 N in NXD25 (*n* = 19) and 163.9 N in SYH (*n* = 17). In the F_2_ population (*n* = 167), stem breaking strength ranged from 20.1 to 673.7 N, with a mean of 156.6 N, a variance of 10,406.1, and a standard deviation of 102.0. The trait displayed a broad continuous distribution with obvious transgressive segregation (Figure 2; Table 1 and Table S4), and the distribution showed positive skewness (1.57) and kurtosis (6.70), further supporting quantitative inheritance. Because the recovered parental dataset showed a clearer phenotypic contrast whereas the F_2_ population still showed a long right tail, the extreme upper-tail observations should be interpreted cautiously and ideally confirmed by repeated phenotyping in subsequent work; accordingly, the present QTL-seq analysis was based on the tails of the F_2_ distribution rather than on simple parental contrasts.

### 2.3. Whole-Genome Resequencing Summary

**Figure 3:**
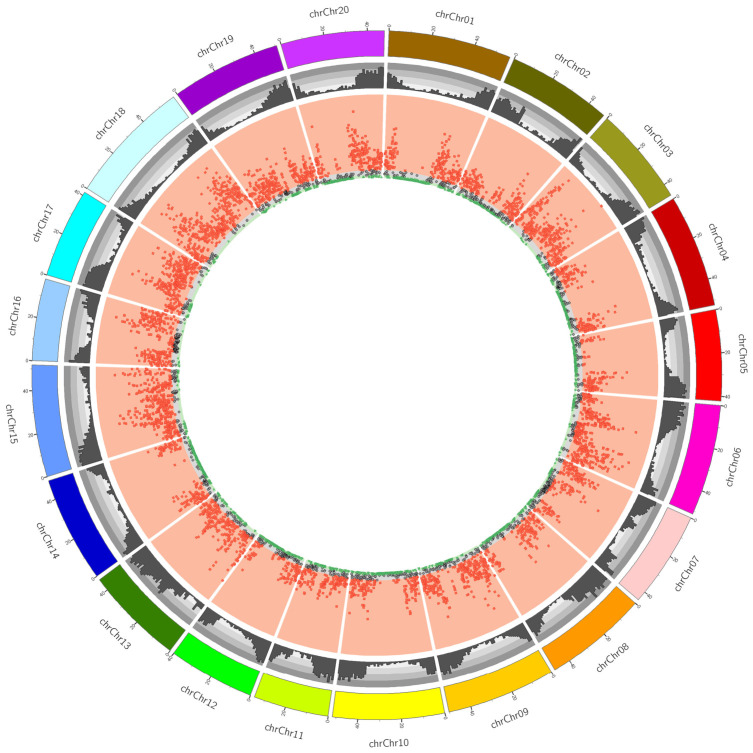
Circular plot summarizing chromosome distribution and genome-wide resequencing features of the soybean population.

Whole-genome resequencing generated 33.85, 37.99, 30.34, and 28.84 Gb of clean data for NXD25, SYH, the HR (high-resistance) pool, and the LR (low-resistance) pool, respectively. Genome coverage ranged from 93.67% to 94.65%, and mapping rates were all above 96.85%. The average sequencing depth reached 31.24× for NXD25, 38.65× for SYH, 27.38× for the HR pool, and 23.64× for the LR pool. A total of 2,340,588 SNPs and 2,295,213 SNPs were detected in the HR and LR pools, respectively. After low-quality variants were removed, 1,085,714 SNPs were retained for subsequent QTL-seq analysis (Figure 3; Table 2).

### 2.4. QTLs Associated with Stem Breaking Strength

**Figure 4:**
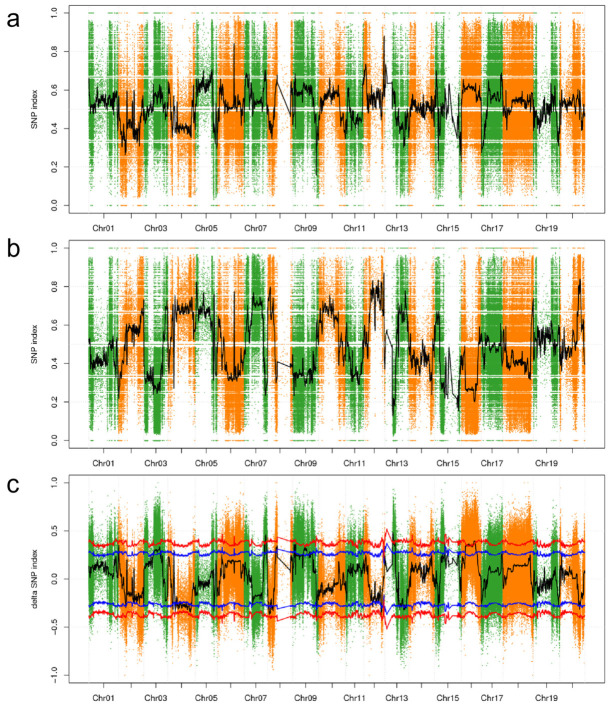
Manhattan plots of QTL-seq mapping for soybean stem breaking strength based on SNP-index analysis. (**a**) Fitted curve of the HR pool; (**b**) fitted curve of the LR pool; (**c**) genome-wide distribution of Δ(SNP-index).

Sliding-window analysis of SNP-index values in the two bulks revealed clear genomic regions associated with stem breaking strength. A window size of 1000 kb and a step size of 100 kb were selected because more than 90% of windows contained at least 10 SNPs. In total, 21 QTL intervals were detected across chromosomes 2, 3, 4, 5, 7, 8, 9, 12, 13, 15, 16, 19, and 20 at the 95% confidence level. Among the QTLs with positive effects on stem breaking strength, 11 intervals were detected on chromosomes 2, 3, 4, 7, 8, 9, 13, 15, and 16. Three major peaks on Chr07, Chr13, and Chr16 exceeded the 99% confidence threshold and were designated qBR7.2, qBR13.1, and qBR16.1. Their physical intervals were 4.90, 5.80, and 22.10 Mb, respectively (Figure 4; Table 3).

### 2.5. Candidate Gene Prioritization Within the Three Major QTL Intervals

To narrow the candidate region, genes within the three major positive QTL intervals were screened based on large-effect sequence variation. Within these intervals, nine genes carried nonsynonymous SNPs and 64 genes harbored frameshift mutations, premature termination codons, or stop-loss mutations. In total, 73 genes were retained as preliminary candidates for the regulation of stem breaking strength, and the full large-effect variant list is provided in Table S1.

By integrating large-effect variation with public SoyBase/SoyOmics expression information, marker localization, and predicted gene functions, nine candidate genes were retained for downstream evaluation. These included six expression-supported genes (*Glyma.16G111300*, *Glyma.13G066200*, *Glyma.16G079300*, *Glyma.16G078700*, *Glyma.16G100200*, and *Glyma.16G119500*) and three marker-linked genes (*Glyma.07G220800*, *Glyma.13G050400*, and *Glyma.16G087300*) located within the three major QTL intervals. The combined annotation and expression evidence for the six expression-supported genes is summarized in Table S3, whereas the three marker-linked genes were additionally highlighted on the basis of InDel-marker development and root/stem-preferential expression evidence from SoyOmics.

### 2.6. Validation of Major QTLs by Co-Dominant InDel Markers

**Figure 5:**
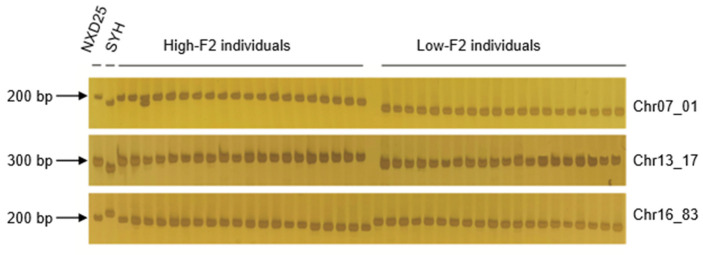
Electrophoretic validation of three co-dominant InDel markers in the parents NXD25, SYH, and selected extreme F_2_ individuals. Lanes: NXD25, high-stem-breaking-strength parent; SYH, low-stem-breaking-strength parent; high-breaking-strength F_2_ individuals; and low-breaking-strength F_2_ individuals. NXD25 and SYH were used as parental genotype references for judging the banding patterns of the selected F_2_ individuals, and the expected product sizes are listed in Table 4. A DNA ladder was not included in the original validation photograph because the comparison focused on parental and extreme-individual band types.

The physical positions of the InDel markers were determined from whole-genome resequencing variant calls against the Wm82.a2 reference genome. To validate the QTL-seq results and support the major regions, 88 primer pairs were designed from exon or intron sequences of 73 genes carrying large-effect variants. After screening, three co-dominant InDel markers linked to stem breaking strength were obtained: Chr07_01 (*Glyma.07G220800*), Chr13_17 (*Glyma.13G050400*), and Chr16_83 (*Glyma.16G087300*) (Table 4). These markers were located within qBR7.2, qBR13.1, and qBR16.1, respectively, and produced clear and stable polymorphic bands between NXD25 and SYH. In the selected extreme F_2_ individuals, the banding patterns were consistent with the high- or low-breaking-strength parental genotypes. Specifically, high-breaking-strength F_2_ materials carried bands matching NXD25, whereas low-breaking-strength materials carried bands matching SYH. The original gel photograph was interpreted by direct comparison with the parental control lanes; no separate DNA ladder was included in that photograph. The expected product sizes for each marker are provided in Table 4. Therefore, these markers should be regarded as preliminary resources that require full-population validation before use as predictive markers (Figure 5).

### 2.7. qRT-PCR Validation of Candidate Genes

**Figure 6:**
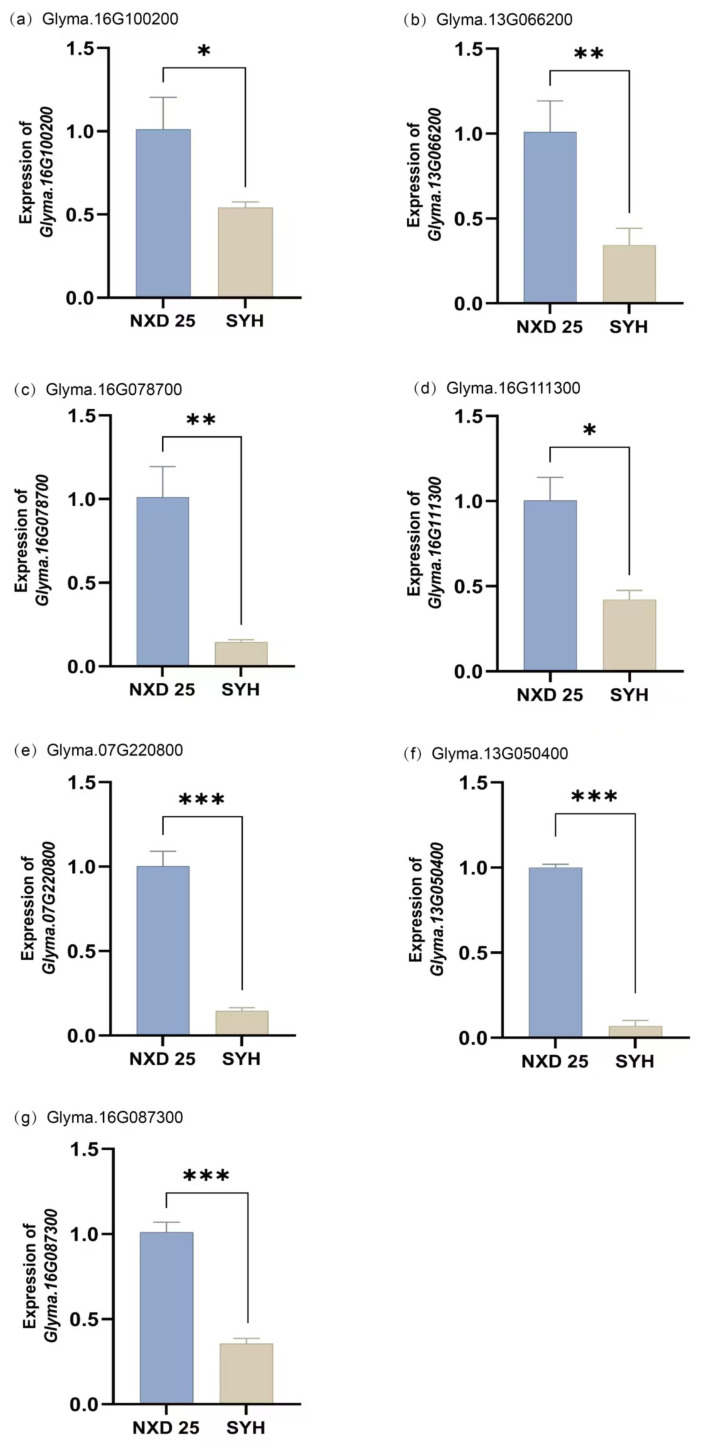
qRT-PCR validation of selected candidate genes in parental stem samples. The upper panel shows seven genes (**a**–**g**) examined in parental stem samples corresponding to the transcriptome-sequencing materials. Bars represent mean ± SD of three biological replicates. Significance labels correspond to the pairwise comparisons indicated in each panel (* *p* < 0.05, ** *p* < 0.01, *** *p* < 0.001; ns, not significant).

qRT-PCR assays were conducted in two steps. First, seven candidate genes were examined in parental stem samples corresponding to the transcriptome-sequencing materials (Figure 6a–g, upper panel). In this assay, *Glyma.16G100200*, *Glyma.13G066200*, *Glyma.16G078700*, *Glyma.16G111300*, *Glyma.07G220800*, *Glyma.13G050400*, and *Glyma.16G087300* all showed higher relative expression in NXD25 than in SYH.

**Figure 7:**
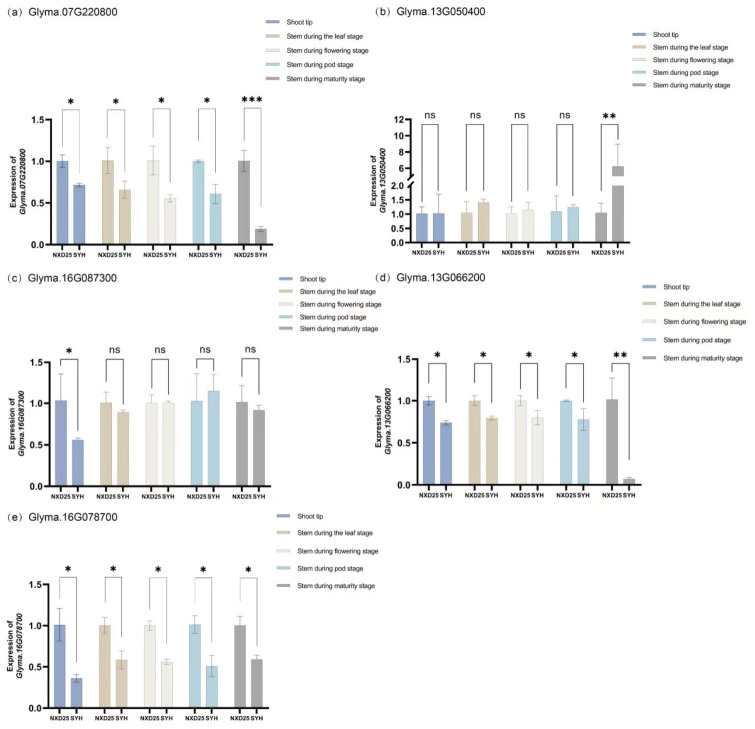
Tissue- and stage-specific expression patterns of five candidate genes in shoot tips and stems at the leaf, flowering, pod, and maturity stages. Panels (**a**–**e**) correspond to different candidate genes. Bars represent mean ± SD of three biological replicates, and significance labels correspond to the pairwise comparisons indicated in each panel (* *p* < 0.05, ** *p* < 0.01, *** *p* < 0.001; ns, not significant). Because the expression ranges differ among genes and tissues, *y*-axis values should not be directly compared across panels.

Second, five genes were examined in shoot tips and stems sampled at the leaf, flowering, pod, and maturity stages (Figure 7a–e, lower panel). *Glyma.07G220800*, *Glyma.13G050400*, *Glyma.16G087300*, *Glyma.13G066200*, and *Glyma.16G078700* showed tissue and stage-dependent expression patterns. Genes not included in the developmental validation were omitted because their expression abundance was low or unstable in the corresponding samples.

### 2.8. Transcriptome-Assisted Refinement of Candidate Genes

**Figure 8:**
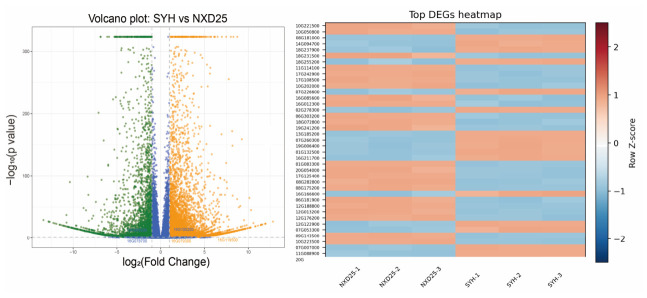
Transcriptome-assisted refinement of candidate genes. (**Left**) volcano plot showing global differential expression between SYH and NXD25. (**Right**) heatmap of representative top differentially expressed genes across the six RNA-seq libraries.

**Figure 9:**
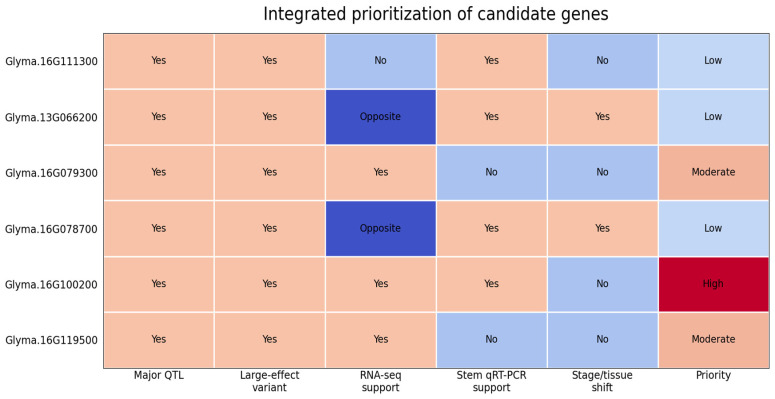
Integrated prioritization of six candidate genes with full gene names: *Glyma.16G111300*, *Glyma.13G066200*, *Glyma.16G079300*, *Glyma.16G078700*, *Glyma.16G100200*, and *Glyma.16G119500*.

Transcriptome sequencing generated an average of 6.95 Gb of clean data per sample, with 44,376 expressed genes detected across the six libraries. The genome mapping rate ranged from 74.04% to 97.96%. Correlation analysis and PCA indicated good repeatability within each genotype and clear separation between NXD25 and SYH. Based on the DESeq2 criteria, 9617 genes were differentially expressed between the two parents, including 6125 up-regulated and 3492 down-regulated genes in SYH relative to NXD25. GO enrichment was dominated by chloroplast- and thylakoid-related terms, whereas KEGG enrichment highlighted pathways such as phenylpropanoid biosynthesis, photosynthesis, plant-pathogen interaction, and ascorbate and aldarate metabolism. When the RNA-seq dataset was overlaid with the three major BSA-Seq intervals, the six expression-supported candidate genes showed distinct expression patterns (Table 5 and Table S2; Figure 8). According to the NXD25/SYH log_2_FC values in Table 5, *Glyma.16G119500* showed the clearest up-regulation in NXD25, while *Glyma.16G079300* and *Glyma.16G100200* also displayed higher transcript abundance in NXD25. In contrast, *Glyma.13G066200* and *Glyma.16G078700* showed relatively higher transcript abundance in SYH in the RNA-seq comparison, which differed from the parental stem qRT-PCR assay but was partly consistent with the tissue/developmental assay. *Glyma.16G111300* showed extremely low transcript abundance in the RNA-seq dataset and was therefore retained only as a provisional candidate on the basis of positional and sequence-variation evidence rather than transcript abundance (Figure 9). In parallel, the three marker-linked genes *Glyma.07G220800*, *Glyma.13G050400*, and *Glyma.16G087300* were retained as high-priority positional candidates because they resided within the major QTL intervals, generated informative InDel markers, and showed root/stem-preferential expression in public SoyOmics data. Overall, a total of nine genes were retained for integrated evaluation, among which, the three marker-linked genes were further highlighted for downstream validation, while *Glyma.16G100200* remained a stable expression-supported candidate in the NXD25-up-regulated group.

## 3. Discussion

Improving stem mechanical strength is a practical route to enhancing soybean lodging resistance, particularly in dense planting and intercropping systems, where lodging reduces yield stability and harvest efficiency [1,20]. The anatomical observations in this study indicate that stronger stems are associated with thicker mechanical tissues, better-developed vascular bundles, and more compact internal organization [7,8,9,10,11]. These features are consistent with lodging-resistance traits reported in other crops and provide a phenotypic basis for interpreting the QTLs identified here [22]. The F_2_ population size and the number of individuals per bulk were sufficient for preliminary BSA-Seq mapping, but larger populations and larger bulks would improve mapping resolution in future studies.

The BSA-Seq strategy used here rapidly identified multiple genomic regions associated with stem breaking strength, and three major QTLs on Chr07, Chr13, and Chr16 exceeded the 99% confidence threshold. Among them, qBR16.1 showed the largest Δ(SNP-index) value and may represent an important hotspot for regulating stem mechanical strength [23,24]. Although the intervals remain broad, this approach efficiently narrowed the search space and provided a framework for subsequent fine mapping. The recovered phenotyping dataset showed a clear parental difference, whereas the F_2_ population displayed substantial transgressive segregation, suggesting that the trait is influenced by recombination among multiple loci. Because the upper-tail values were much higher than the parental means [25], these extreme observations should be confirmed by repeated phenotyping. Comparable studies in rice and maize have also identified stem-mechanical or stalk-strength loci using QTL mapping, QTL-seq, or related bulked-segregant strategies [26,27,28]. The qBR16.1 interval spanned 22.10 Mb and contained numerous genes, but the multi-omics strategy reduced the list to nine prioritized candidates for subsequent validation.

A major strength of this study is the integration of sequence variation, RNA-seq, qRT-PCR, and marker evidence to prioritize biologically plausible candidates. RNA-seq identified 9617 differentially expressed genes between the two parents and highlighted phenylpropanoid biosynthesis, a pathway closely related to lignin metabolism and stem mechanical strength [10,11,29,30]. GO enrichment also pointed to coordinated differences in photosynthetic and chloroplast-associated functions, suggesting that stem-strength variation may be embedded within broader developmental and metabolic divergence between the parents [31]. Within the integrated nine-gene framework, six genes were supported mainly by transcriptome and qRT-PCR evidence [32], whereas *Glyma.07G220800*, *Glyma.13G050400*, and *Glyma.16G087300* were retained because they generated informative InDel markers and showed root/stem-preferential expression in public SoyOmics data. *Glyma.16G100200* showed relatively stable higher expression in NXD25 across RNA-seq and parental stem qRT-PCR assays. In contrast, *Glyma.13G066200* and *Glyma.16G078700* showed different expression directions among the RNA-seq comparison, parental stem qRT-PCR assay, and developmental assay, indicating stage- and tissue-dependent regulation rather than a constitutive association with the trait. *Glyma.16G111300* showed extremely low transcript abundance in RNA-seq and should be regarded as a provisional candidate pending additional validation.

The development of three co-dominant InDel markers further increases the practical value of this study. These markers were designed from polymorphic loci within the mapped regions and showed phenotype-consistent banding patterns in selected extreme materials. In the gel validation, the parental lanes were used as genotype references: Chr07_01, Chr13_17, and Chr16_83 amplified different polymorphic bands in NXD25 and SYH, and the selected extreme F_2_ individuals showed band types consistent with their corresponding parental phenotype groups. Because the linked genes also displayed root/stem-preferential expression in public SoyOmics data [33], the marker information provides an additional line of evidence for candidate prioritization. However, the current validation was limited to extreme F_2_ individuals [34]. These markers should therefore be viewed as preliminary resources for further verification, not as fully validated predictive tools for quantitative performance across the entire population.

The three marker-linked genes were not included among the six expression-supported genes because they were prioritized on the basis of InDel polymorphism, physical location, and root/stem-preferential expression rather than transcript abundance alone [35]. Several limitations should be noted. First, the F_2_ population size was modest for BSA-Seq analysis, which reduced mapping resolution and contributed to the relatively broad intervals identified here. Second, the sliding-window parameters were set to 1000 kb with a 100 kb step because smaller windows produced too many regions with insufficient informative SNPs; however, this choice also widened the final candidate intervals [23,24]. Third, marker validation was restricted to selected extreme individuals rather than the full F_2_ population. Finally, some candidate genes showed inconsistent expression directions among RNA-seq, parental stem qRT-PCR, and developmental qRT-PCR datasets. These results are compatible with strong spatiotemporal regulation, but they also indicate that fine mapping, larger population-based marker testing, repeated phenotyping of extreme individuals, and stage-matched expression analyses are required before causal genes can be identified with confidence [26,27].

## 4. Materials and Methods

### 4.1. Plant Materials and Population Construction

Nanxiadou25 (NXD25) is an elite soybean line with high stem breaking strength, strong lodging resistance, high yield, and wide adaptability. SYH is a local landrace with low stem breaking strength and weak lodging resistance. A cross between NXD25, an elite soybean line developed by the Nanchong Academy of Agricultural Sciences, and the local landrace Shiyuehuang (SYH) was made in August 2017. F_1_ seeds were advanced in Hainan in December 2017, and individual F_2_ seeds were harvested in April 2018. In June 2018, the two parents and the F_2_ population were grown at the Yingxi experimental station of the Nanchong Academy of Agricultural Sciences under conventional field management. All parental and F_2_ materials were maintained under the same agronomic conditions to minimize environmental heterogeneity among samples. At the stage when the first trifoliate leaf was fully expanded, individual plants were tagged and sampled for later molecular analyses [36].

### 4.2. Histological Observation of Stem Cross Sections

To compare stem anatomical characteristics between the parents, stem segments were fixed in 4% paraformaldehyde and subjected to routine paraffin sectioning. Samples were dehydrated in a graded ethanol series, cleared with xylene, embedded in paraffin, and sectioned at 4 μm thickness. Sections were deparaffinized, stained with safranin and fast green, mounted with neutral resin, and scanned using a 3DHISTECH Pannoramic SCAN digital slide scanner (NIKON ECLIPSE E100/NIKON DS-U3, Tokyo, Japan). Images were collected at low and high magnification for comparative observation of the xylem, phloem, cambium, and pith tissues [37]. Images were collected at 4.0× (low magnification) and 20.0× (high magnification) using a 3DHISTECH Pannoramic SCAN digital slide scanner.

### 4.3. Measurement of Stem Breaking Strength and Bulk Construction

After seed maturation, single-plant stems were collected and naturally air-dried to constant weight. Stem breaking strength was measured at 15 cm above the cotyledonary node using a plant stem strength tester (YY-1; Zhejiang TOP Yunnong Technology Co., Ltd., Hangzhou, China). Based on the phenotypic values, the parental bulks were each constructed from five plants showing stable stem breaking strength. For the F_2_ population, plants were ranked from highest to lowest stem breaking strength, and the top 30 and bottom 30 individuals were selected to construct the high-resistance (HR) and low-resistance (LR) bulks, respectively. Genomic DNA was extracted from each plant using the CTAB method [38] and pooled in equal amounts within each bulk. Whole-genome resequencing was performed on the Illumina HiSeq × Ten PE150 platform. The target sequencing depth was approximately 30× for each parent and 10× for each bulk.

### 4.4. Read Mapping, Variant Calling, and QTL-Seq Analysis

Raw reads were filtered to obtain high-quality clean reads and aligned to the soybean reference genome Wm82.a2 using Burrows-Wheeler Aligner (bwa-0.7.19 (r1273)). SNPs and InDels were called using Genome Analysis Toolkit (GATK 4.2.6.0) with the Wm82.a2 reference genome (https://phytozome-next.jgi.doe.gov/info/Gmax_Wm82_a2_v1) (accessed on 30 September 2025). Only loci that were homozygous but polymorphic between the two parents and had sequencing depth greater than 5 in both parents were retained as informative variants [12,13]. SNP-index values were calculated for the two bulks using a sliding-window approach with a window size of 1000 kb and a step size of 100 kb. Sites with SNP-index values below 0.3 in both bulks or sequencing depth below 7 were excluded. Preliminary trials with smaller windows produced too many regions with insufficient informative SNPs, and the 1000 kb window therefore provided a more stable genome-wide profile for the present dataset. The Δ(SNP-index) values between the bulks were estimated for each site and window. Significance thresholds for the 95% and 99% confidence intervals were obtained by 1000 permutation tests, and peak regions above the threshold were defined as candidate QTL intervals [15,16].

### 4.5. Candidate Gene Prioritization and InDel Marker Development

Genes located within the three major QTL intervals were screened on the basis of large-effect sequence variation. Particular attention was given to genes carrying nonsynonymous SNPs with high Δ(SNP-index) values and to genes containing frameshift insertions, frameshift deletions, premature stop codons, or stop-loss mutations. Public transcriptome annotation from SoyBase and predicted gene functions were further used to prioritize candidate genes with preferential expression in roots and stems. Based on QTL-seq results, InDel loci located near major peaks with |Δ(SNP-index)| ≥ 0.3 were selected for marker development. InDel positions were determined from genome resequencing data and Wm82.a2 reference coordinates. Primers were designed using Primer Premier 5 and NCBI Primer-BLAST (https://www.ncbi.nlm.nih.gov/tools/primer-blast/) (accessed on on 30 October 2025). During gel validation, the NXD25 and SYH parental PCR products were run as genotype reference controls beside extreme F_2_ samples. Band types were assigned by comparison with these parental controls, and product-length differences were interpreted together with the expected amplicon sizes listed in Table 4.

### 4.6. qRT-PCR Analysis

qRT-PCR was used to validate selected candidate genes identified by BSA-Seq. Total RNA was extracted from parental stem tissues and from additional shoot tip and stem samples collected at the leaf, flowering, pod, and maturity stages. The primers listed in Table 6. Reverse transcription and amplification were performed according to the manufacturer’s protocol using a one-step qRT-PCR kit, with ACT11 as the internal reference gene. Relative transcript abundance was calculated using the 2^−ΔΔCt^ method with ACT11 as the internal reference gene. Relative expression data are presented as mean ± SD, and pairwise comparisons between parents were interpreted based on the significance labels shown in the corresponding figures.

### 4.7. Transcriptome Sequencing and Integrative Analysis

For transcriptome analysis, six libraries were constructed from the two parents (NXD25-1, NXD25-2, NXD25-3, SYH-1, SYH-2, and SYH-3) and sequenced on an Illumina platform using a PE150 strategy (Illumina NovaSeq Xplus, San Diego, CA, USA). Raw reads were filtered using fast preprocessor (fastp v1.2.0), and clean reads were aligned to the soybean reference genome Glycine_max_v2.1 (release-57) with STAR 2.7.11b. Gene-level read counts were obtained with featureCounts and normalized expression was represented as FPKM. Sample correlation analysis and principal component analysis were performed to assess repeatability and group separation. Differential expression analysis between SYH and NXD25 was carried out using DESeq2, with |log_2_(fold change)| > 1 and *p*adj < 0.05 used as the significance threshold. GO and KEGG enrichment analyses of DEGs were then interpreted together with the BSA-Seq candidate intervals to refine the candidate gene list. For consistency with the fixed qRT-PCR figures, the resistant parent is abbreviated as NXD25 in the manuscript text; the technical RNA-seq sample IDs generated by the sequencing provider remain NXD25-1 to NXD25-3, and the original comparison label SYH_vs_NXD25 is retained where it refers to analysis output.

## 5. Conclusions

Lodging resistance is a critical target for soybean breeding aimed at stable yields in dense planting and intercropping systems, and stem breaking strength is a key determinant of lodging tolerance. In this study, an F_2_ population consisting of 167 individuals derived from the cross NXD25 × SYH was used to identify QTLs and candidate genes for stem breaking strength by BSA-Seq and QTL-seq. A total of 21 QTL intervals were detected, among which, qBR7.2, qBR13.1, and qBR16.1 exceeded the 99% confidence threshold. By integrating large-effect variant analysis, transcriptome profiling, qRT-PCR validation, and InDel marker development, nine candidate genes were prioritized, and *Glyma.07G220800*, *Glyma.13G050400*, and *Glyma.16G087300* were proposed as high-priority candidates for functional validation. In addition, Chr07_01, Chr13_17, and Chr16_83 showed stable and phenotype-consistent polymorphism in selected extreme materials, with parental lanes used as genotype references. These results provide useful QTLs, candidate genes, and preliminary molecular markers for marker-assisted breeding of lodging resistance in soybean.

## Figures and Tables

**Table 1:** Phenotypic statistics of stem breaking force in the parents and the F_2_ population.

Trait	NXD25	SYH	Range	Mean	Variance	SD	CV	Skewness	Kurtosis
Stem breaking strength (N)	257.3	163.9	20.1–673.7	156.6	10,406.1	102.0	0.652	1.57	6.70

**Table 2:** Summary of resequencing quality and variant statistics for the two parents and the two bulks.

Sample	Clean Bases (Gb)	Total Reads (M)	Q20 (%)	Mapped Reads (%)	Genome Coverage (%)	Total SNPs	Nonsynonymous SNVs	Synonymous SNVs	Total InDels	Frameshift Indels	Non-Frameshift Indels
NXD25	33.85	225.68	94.48	97.36	94.01	1,633,184	35,748	27,442	304,477	2297	1688
SYH	37.99	253.31	94.20	96.88	93.67	1,783,780	37,175	28,171	324,902	2333	1023
HR pool	30.34	202.24	94.09	96.85	94.65	2,340,588	50,922	38,778	410,984	2989	2372
LR pool	28.84	192.27	94.26	96.85	94.22	2,295,213	50,104	38,281	402,332	2942	2340

**Table 3:** QTL intervals associated with stem breaking strength identified by QTL-seq.

Region	QTL	SNP Number	InDel Number	SNP-Index (LR Pool)	SNP-Index (HR Pool)	Δ (SNP-Index)	Interval Size (Mb)
Chr02:370-998591	qBR2.1	2051	996	0.2259	0.5538	0.3279	1.00
Chr02:14000442-14097489	qBR2.2	224	116	0.5709	0.2211	−0.3499	0.10
Chr03:17616536-32597049	qBR3.1	48,554	6935	0.2764	0.5753	0.2989	14.98
Chr04:8511986-45598101	qBR4.1	11,348	3757	0.6809	0.3821	−0.2988	37.09
Chr04:11200942-11499718	qBR4.2	3	283	0.5214	0.3822	−0.1392	0.30
Chr05:35701079-36199988	qBR5.1	164	615	0.6912	0.3833	−0.3079	0.50
Chr07:15901469-16199912	qBR7.1	302	179	0.7345	0.4428	−0.2917	0.30
Chr07:38303811-43199546	qBR7.2	3655	1216	0.3226	0.6529	0.3303	4.90
Chr08:2600019-11099893	qBR8.1	8314	4743	0.7151	0.4035	−0.3116	8.50
Chr08:15809698-18499784	qBR8.2	5744	2309	0.3193	0.6295	0.3103	2.69
Chr09:2900031-37398314	qBR9.1	14,638	4457	0.3412	0.6234	0.2823	34.50
Chr09:45501079-46998248	qBR9.2	950	586	0.6115	0.3087	−0.3029	1.50
Chr12:16064263-17468379	qBR12.1	17	217	0.8259	0.5539	−0.2719	1.40
Chr12:32506773-34398406	qBR12.2	277	347	0.8028	0.4653	−0.3375	1.89
Chr13:14400478-20197251	qBR13.1	4040	2831	0.1811	0.5804	0.3993	5.80
Chr13:28600171-41398386	qBR13.2	1579	3947	0.7135	0.4204	−0.2932	12.80
Chr15:7102356-7795013	qBR15.1	222	488	0.6303	0.3405	−0.2898	0.69
Chr15:19902772-25564559	qBR15.2	4	317	0.1842	0.3995	0.2152	5.66
Chr16:7300151-29399886	qBR16.1	51,107	9304	0.2547	0.6061	0.3513	22.10
Chr19:48303770-48738399	qBR19.1	1	164	0.7308	0.4118	−0.3191	0.43
Chr20:37400040-37899870	qBR20.1	437	422	0.7744	0.4883	−0.2861	0.50

**Table 4:** Co-dominant InDel markers linked to the three major QTLs.

Gene with LEV	InDel Position	Marker	Primer Sequence (5′-3′)	Tm (°C)	Expected PCR Product Length (bp)
*Glyma.07G220800*	39567744	Chr07_01	F: GTCCAGGGCACTTGTCG R: GCTCCCACTTTAGGGTAGA	54.0/53.9	259/218
*Glyma.13G050400*	14654278	Chr13_17	F: TCAGATACAAATACGGCTAC R: TGAGATGTGATACGTGAGTT	48.0/47.4	364/331
*Glyma.16G087300*	10989731	Chr16_83	F: GAGGATGAAGCTGCTCCAAG R: TGACGCTGCAATGACTGAAC	57.3/57.5	207/223

**Table 5:** Differential expression features of candidate genes located within the three major QTL intervals.

Gene ID	SYH Mean FPKM	NXD25 Mean FPKM	log_2_FC (NXD25/SYH)	*p* Value	RNA-Seq Trend/DEG Status
*Glyma.16G111300*	0.000	0.000	NA	NA	extremely low/not evaluated
*Glyma.13G066200*	14.084	11.863	−0.314	6.07 × 10^−7^	SYH higher/non-DEG
*Glyma.16G079300*	0.920	3.021	1.621	2.87 × 10^−2^	NXD25 higher/DEG
*Glyma.16G078700*	1.649	1.133	−0.616	4.03 × 10^−1^	SYH higher/non-DEG
*Glyma.16G100200*	1.981	3.308	0.667	1.02 × 10^−4^	NXD25 higher/non-DEG
*Glyma.16G119500*	0.000	0.121	4.307	2.36 × 10^−2^	NXD25 higher/DEG

Note: log_2_FC, log_2_(fold change); FC, fold change. DEG status was assigned using |log_2_FC| > 1 and *p*adj < 0.05. Genes with weak fold changes or extremely low transcript abundance were treated as non-DEGs or provisional candidates, even when a nominal *p* value was available.

**Table 6:** Primers used for qRT-PCR validation of selected candidate genes.

Gene ID	Primer Name	Direction	Sequence (5′–3′)
*Glyma.13G066200*	qGm13G066200-F2	F	AACAGCCAGATACTAATGGAA
	qGm13G066200-R2	R	GGACCTGATAGTGTCACTGGT
*Glyma.16G078700*	qGm16G078700-F2	F	GCTGCTAATAATGTTGCTGAT
	qGm16G078700-R2	R	CACAATCTATATTCAGCCAGT
*Glyma.16G100200*	qGm16G100200-F1	F	GTAGACGAGTGCTCTAAAATG
	qGm16G100200-R1	R	GATGGTGGGAATGGTGCA
*Glyma.16G111300*	qGm16G111300-F2	F	CGGAGGATGTTGTGAGGAGG
	qGm16G111300-R2	R	CTGGTGAAGCAGGCCAATGGA
*Glyma.16G119500*	qGm16G119500-F1	F	TCCAATTAGGCCTTGCATACA
	qGm16G119500-R1	R	AATATTCGGGATTTCGGGTAA
*Glyma.16G079300*	qGm16G079300-F1	F	ATTCATGTTAAATCCCGGGAC
	qGm16G079300-R1	R	CAATGTCATGTTGGTTATCATG

## Data Availability

The transcriptome sequencing data generated in this study have been deposited in the NCBI Sequence Read Archive (SRA) under BioProject accession PRJNA1455227. The final phenotyping dataset, marker information, and processed candidate-gene annotation and expression summaries are included in the article and Supplementary Materials. The original BSA-Seq resequencing data are available from the corresponding authors upon reasonable request.

## Supplementary Materials

The following supporting information can be downloaded at: https://www.mdpi.com/article/10.3390/plants15111610/s1, Table S1. Genes carrying large-effect SNP/InDel variants within the Annotation Information for SNPs InDel in the Negative Threshold Peak Region; Table S2. Genes carrying large-effect SNP/InDel variants within the three major positive-effect QTL intervals qBR7.2, qBR13.1, and qBR16.1; Table S3. Integrated annotation and expression summary of the six expression-supported candidate genes; Table S4. Final phenotyping dataset used for stem breaking strength analysis.
